# Medication nonadherence - definition, measurement, prevalence, and causes: reflecting on the past 20 years and looking forwards

**DOI:** 10.3389/fphar.2025.1465059

**Published:** 2025-03-07

**Authors:** Sarah C. E. Chapman, Amy H. Y. Chan

**Affiliations:** ^1^ Centre for Adherence Research and Education, Institute of Pharmaceutical Science, King’s College London, London, United Kingdom; ^2^ School of Pharmacy, Faculty of Medical and Health Sciences, The University of Auckland, Auckland, New Zealand

**Keywords:** medication adherence, measurement, definition, causes, prevalence

## Abstract

In 2003, Sabate’s World Health Organisation report defined medication nonadherence as a phenomenon where individuals’ behaviour does not correspond to prescribed treatment recommendations from their healthcare provider. This concept of nonadherence evolved beyond a categorisation of patients as adherent or nonadherent. Rather, nonadherence varies within the same individual and treatment over time, and between treatments and individuals. The type and patterns of nonadherence are key determinants of outcome with individuals with the same percentage nonadherence having different outcomes depending on their pattern of nonadherence. Often the poorest clinical outcomes occur in individuals who do not initiate medication or discontinue early, but much of the nonadherence literature remains focused on implementation. This paper provides a nuanced discussion of nonadherence which has been enabled in part by the growing availability of technologies such as electronic nonadherence monitors, new biomarkers for adherence and greater access to ‘big data’ (e.g., on prescription refills). These allow granular assessment of nonadherence that can be linked with biophysical markers captured using technologies such as wearables. More validated self-report measures have also become available to profile nonadherence in research and practice. Together, in-depth data on dosing and clinical measures provide an opportunity to explore complex interactions between medications, therapeutic effects and clinical outcomes. This variation in measurement and definition means that there is a more fine-grained understanding of the prevalence of nonadherence and a greater recognition of the prevalence of nonadherence, with growing evidence suggesting that approximately a fifth of patients do not initiate treatment, of those initiating treatment approximately 30%–50% of patients do not implement their treatment as prescribed and that, over long follow-up periods in some conditions 80%–100% of patients discontinue. There is potential too to better understand causes of nonadherence. New behavioural models synthesise determinants of nonadherence previously considered separately. Frameworks like the COM-B (considering individual capability, opportunity, and motivation factors) and MACO (focusing on Medication Adherence Contexts and Outcomes) emphasize the multifaceted nature of nonadherence determinants. Greater focus on dynamic processes with interplay between individual, social, and environmental influences is needed. Addressing these complexities could lead to more effective and personalised support for patients.

## 1 Introduction

The landmark 2003 World Health Organisation medication nonadherence report (Sabaté, 2003) begins with discussion of the definition of nonadherence, highlighting the need to go beyond medication and to consider patients as active in generating healthcare recommendations. The authors conclude adherence is “*the extent to which a person’s behaviour – taking medication, following a diet, and/or executing lifestyle changes, corresponds with agreed recommendations from a healthcare provider”*. This definition locates nonadherence behaviour within a person who is in receipt of recommendations rather than within the healthcare provider or system. It implies nonadherence is continuous, rather than easily categorised into “adherent” vs. “nonadherent” behaviour.

**FIGURE 1:**
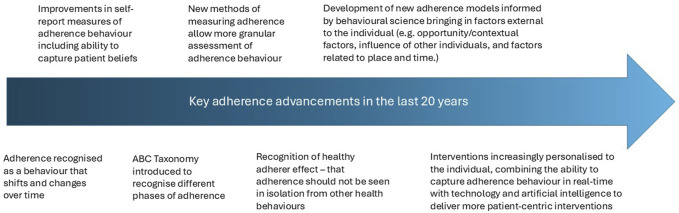
Key advancements in medication adherence in the last 20 years.

This perspective paper will outline key developments across the areas of definition, prevalence, measurement and causes of nonadherence over the last 20 years and discuss future directions in these areas (see Figure 1).

## 2 Developments in definitions of nonadherence: going beyond definitions to taxonomies and processes

Since the WHO adherence report, the definition of adherence has continued to be debated with more recent models including elements of health provider behaviour, and, in the case of medication adherence, splitting adherence into multiple behaviours rather than conceptualising this as a single, consistent behaviour [e.g., (Vrijens et al., 2012; Bartlett Ellis et al., 2023; Chan et al., 2020; Xu et al., 2023)]. For example, digital medication packaging and devices can record the number and timing of medication doses accessed by a patient, providing a more detailed picture of medication-taking over time than traditional measures such as dispensing records (Koledova et al., 2020). Together these changes in measurement have highlighted that adherence can be thought of as multiple behaviours, occurring at different times and places. Some of these behaviours may be performed alone, whereas others are reliant on carers, friends, family, healthcare professionals and healthcare systems (Bartlett Ellis et al., 2023).

Nonadherence as a concept has existed since 400BC when Hippocrates wrote “keep a watch. on the faults of the patients, which often make them lie about the taking of things prescribed. For through not taking disagreeable drinks, purgative or other, they sometimes die” (Brown and Bussell, 2011). This quote summarises tenets of nonadherence that are applicable today - nonadherence is common; patients can conceal nonadherence, and nonadherence can negatively affect health including mortality (Simpson et al., 2006). By the 1970s, nonadherence research was established though referred to as non-compliance research (Becker and Maiman, 1975). The importance of involving patients in treatment decisions was recognised further since this time, and the terminology of concordance was developed in the 2000s to reflect the agreement process between the prescriber and patient. However, uptake of the term concordance has not been far-reaching (Hugtenburg et al., 2013). In 2012, Vrijens et al. (2012) established the first taxonomy to describe (non)adherence behaviour and following this, the EMERGE guideline was published to standardise reporting of nonadherence research using this taxonomy (De Geest et al., 2018). These events have shaped the definition of adherence over the last decade. Rather than considering adherence as a static patient characteristic where patients are classified as ‘adherent’ or “nonadherent” based on an assessment at a single point in time, adherence is now understood to be a behaviour which can differ both between and within the same individual over time (Horne, 2006). Reviews of reviews (Gast and Mathes, 2019) have also shown that it is difficult to use overt stable patient characteristics such as socio-demographic factors, traits, illness or treatment characteristics to predict nonadherence; rather nonadherence is frequently driven by treatment beliefs and illness perceptions (Horne et al., 2013; Foot et al., 2016). For example, being prescribed a treatment which is perceived as a newer medication within the same therapeutic class is associated with a 2.5% reduction in nonadherence for every 10 years increase in medication ‘newness’ - independent of treatment regimen, condition or patient characteristics (Blankart and Lichtenberg, 2020).

Nonadherence should ideally be viewed holistically considering other health behaviours (Steiner, 2012). Adherence to even placebo medication has mortality benefits (odds of dying 0.56 with adherence to placebo *versus* 0.55 for adherence to medication compared to nonadherence) (Simpson et al., 2006). This is known as the ‘Healthy Adherer Effect’ (Chewning, 2006). The psychological basis for the Healthy Adherer Effect has been less well-elucidated. Potentially it in part occurs because different health behaviours do not occur in isolation and can interact with one another and be influenced by common factors within and outside of the individual (Steiner, 2012).

Despite increasing research supporting the need to conceptualise nonadherence as a behaviour and not a non-modifiable trait (Horne et al., 2019; Horne et al., 2018), there continues to be research that characterises patients as adherent or nonadherent. With the complexities of nonadherence as a behaviour, we propose that there is a need to move towards more granular conceptualisation of nonadherence and to explore why and how nonadherence changes over time, and whether there are different factors that affect the different stages of nonadherence differently.

## 3 Prevalence of nonadherence

The widely cited statistic on the prevalence of nonadherence states that approximately 30%–50% of patients do not take their prescribed medication as recommended (Sabaté, 2003). However, the reality is that the rate of nonadherence is likely to vary across patient groups, medications, measurement methods, how strict a definition of nonadherence is used, and the timing and time period an adherence measure covers. Given that increasingly nonadherence is viewed as happening on a continuum (see Section 2 above), estimates of nonadherence prevalence may be inherently flawed as they rely on categorising individuals dichotomously (De Geest et al., 2018). Estimates of the prevalence of nonadherence should therefore refer to the type of nonadherence and the period over which it is assessed. Additionally, it is hypothesised that prevalence estimates that cover a longer period of time or use a stricter cut-off for adherence may be likely to come to the conclusion that a higher proportion of patients are not adherent.

### 3.1 Prevalence of nonadherence to initiating treatment

Much nonadherence research focuses on implementation once treatment is started despite evidence showing that non-initiation of medication (primary nonadherence) is associated with poorer health outcomes including higher mortality rates (Jackevicius et al., 2008) and emergency department visits (Lee et al., 2016). The limited research that exists exploring reasons for non-initiation suggest that the factors that influence patients’ decisions to initiate a medication or not are similar to the factors influencing whether a patient continues to take a medication long-term or stops it prematurely (discontinuation or non-persistence). Part of non-initiation is primary nonadherence, whereby a medication is newly prescribed but then the prescription is not filled at a pharmacy (Fischer et al., 2010). Cheen et al. (2019) systematically reviewed 33 studies and estimated that 17% of patients with six long-term conditions did not collect a newly prescribed medication with rates highest in osteoporosis and hyperlipidaemia (both 25%) and lowest in diabetes mellitus (10%). Studies of patients with a mean age under 65 years old had significantly higher primary nonadherence rates. But, lower primary nonadherence was found in patients aged 19–44 than in children or patients aged over 45 in a recent analysis of 34,243 Canadian primary care patients (Zeitouny et al., 2023) but older patients with polypharmacy were also at increased risk of primary nonadherence. Rates of primary nonadherence are also likely to vary with treatment type and healthcare system factors; Anaba and Arabambi (2022) examined rates at which dermatology patients collected a prescribed medication from a hospital in Lagos, Nigeria, and found 72% topical medications were not collected compared to 23% of oral medications, with more than half of patients who had not collected a medication saying that lack of availability and cost were the reasons for their nonadherence (Anaba and Arabambi, 2022).

**FIGURE 2:**
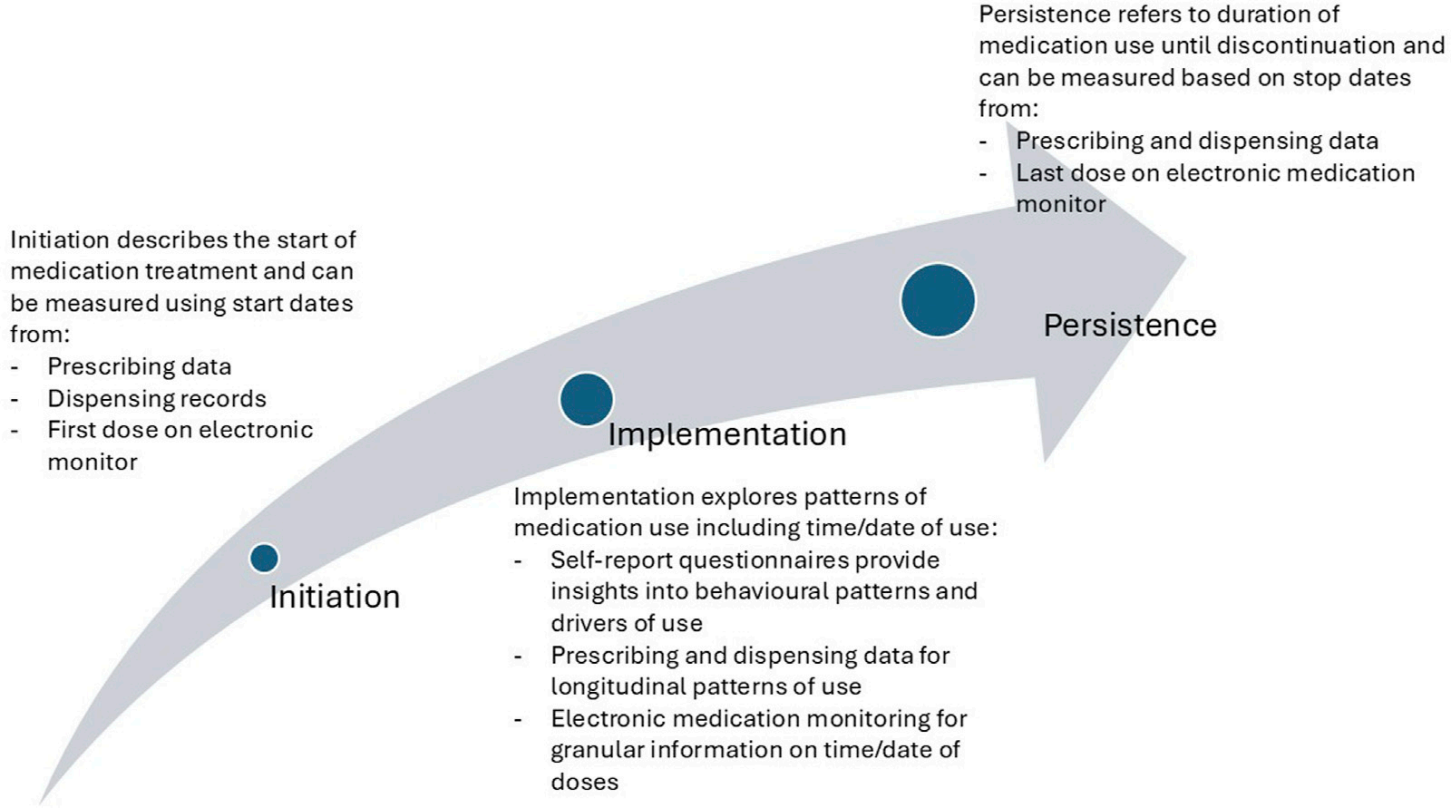
Different stages of adherence and data sources to assess each adherence stage.

Many patients who collect a medication (or have it delivered to them after dispensing) may still not initiate treatment (i.e., take the first dose) (Fischer et al., 2010). Estimates of the number of patients who obtain a prescription but then do not take the first dose are not widely available. Few adherence measures specify whether any dose is taken. Where digital adherence monitors (e.g., MEMs caps) are used to monitor adherence with oral medication in newly treated patients over time, there appear to be low rates of patients with 100% nonadherence (Hebing et al., 2022) but, people participating in research studies in which adherence is monitored may be more likely to initiate treatment than people who are not monitored. Studies of medication waste, such as analysis of medications returned to Dutch community pharmacies (Bekker et al., 2018), report returns of unopened packets, perhaps hinting that not all collected medications may be started. With the difficulties with capturing medication initiation, the true rates of non-initiation may be to accurately measure. Triangulating different data sources such as linking prescribing and dispensing records, along with electronic adherence monitoring, may help provide useful estimates of rates of non-initiation (Figure 2).

### 3.2 Prevalence of implementation nonadherence

Implementation nonadherence is the most frequently assessed and commonly known form of nonadherence, with rates varying widely across patient groups, contexts and medication (Gast and Mathes, 2019; Foley et al., 2021). Implementation nonadherence is most often assessed in relation to number of doses taken, but can encompass timing, amount of medication taken, overuse, and adherence to other instructions (e.g., combination with food/fluid) (Nieuwlaat et al., 2014). Helmy et al. (2019) estimated that 37.9% of patients receiving immunosuppressants after heart transplant did not implement their medication as prescribed, within this 26.2% of patients took their immunosuppressant at a different time from that prescribed, while 17.3% did not take all of their immunosuppressant doses.

Rates of nonadherence are also likely to vary depending on the cut-off used to classify participants as nonadherent and the time period evaluated. Davis et al. (2010) estimated that 61% of patients with Parkinson’s disease took less than 80% of their medication (based on prescription refill data) over a 7 year period. Whereas, Buh et al. (2023) found 37.7% of patients with HIV at a clinic in Cameroon had missed one or more dose of their medication in the last month. When following up people taking antiretroviral treatment for 20 months using electronic monitoring, Wagner et al., found implementation nonadherence rates increased as time progressed (Wagner et al., 2020).

Rates may vary systematically across different contexts or healthcare systems, for example, Mahmood et al. (2022) reviewed 66 studies assessing implementation nonadherence to antihypertensives in Asia, and estimated an overall prevalence of 48% nonadherence, but found wide variation across regions (Mahmood et al., 2021). Relating to healthcare system factors, rates of antihypertensive implementation nonadherence in one cohort from Islamabad were lower in tertiary care patients than primary and secondary care patients and lower in those who had access to free medical care than those who did not (Mahmood et al., 2020). As with all factors that contribute to medication nonadherence, it is important not to overgeneralise or assume simple causation when considering associations between healthcare system and context factors and implementation nonadherence rates. For example, cost-related implementation nonadherence may occur because of medication unaffordability, but may also occur because groups who experience cost-related medication nonadherence may also be at increased risk of depression, which itself is linked to nonadherence (Briesacher et al., 2007; Gonzalez et al., 2011).

Implementation nonadherence is also likely to be higher for treatments that are more difficult to take or access. Okada et al. (2021) review studies estimating the prevalence of nonadherence to intravitreal ocular therapy for macular degeneration, which requires attendance at regular appointments for injections into the eye, and found rates of implementation nonadherence as high as 95.6% (Okada et al., 2021). There is evidence that treatments that involve multiple doses, or are involve complicated dosing instructions also achieve poor implementation rates (Ingersoll and Cohen, 2008).

Interestingly, there is emerging evidence that suggests there may be time-of-day effects on medication adherence with morning doses achieving greater adherence than evening doses. Phillips et al. conducted a study with electronic medication monitors in patients on twice-daily dosing for type 2 diabetes over 1 month and found that patients overall missed fewer morning pills (Phillips et al., 2021). However, the authors did not find that variability in dose timing differed between morning compared to evening. Thus, better morning adherence may not be due to consistency in the timing *per se* of the medication taking, but perhaps the linking of the morning adherence with a particular consistent routine such as morning coffee, which could vary in timing across different days. In contrast, the evenings may be more disrupted where the medication is either not taken at all, or if remembered, was taken at roughly the same time each evening. More research into the role of behavioural patterns and routines on routine medication taking is warranted to explore time-of-day and seasonal effects. Overall, implementation nonadherence can be said to be common but prevalence estimates are highly variable given the variation in conditions, seasonal and timing effects.

### 3.3 Prevalence of non-persistence

Non-persistence, whereby patients stop taking a medication before the time agreed with a healthcare professional is generally believed to increase over time. For example, Joret et al. (2022) found that non-small cell lung cancer patients took an estimated 98% of doses at the beginning of tyrosine kinase inhibitor treatment, but that at around 2 months, nearly half of patients had discontinued treatment and at 4 months more than 60% had discontinued. Hardtstock et al. (2022) evaluated non persistence to long-acting asthma treatments over a 12 months follow-up period and estimated non-persistence at 86.7%. There is some suggestion that non persistence increases with experience of adverse effects, with Fleming et al. (2022) finding that a majority of studies included in a review of adjuvant breast cancer treatment persistence found that patients who reported more adverse effects were more likely to discontinue. Alefan et al. (2022) found that adverse effects were particularly strongly linked to rates of discontinuation when the adverse effects were not anticipated by the patient.

Taking the estimated rates of initiation, implementation and persistence together, it is hypothesised that the often-quoted estimate of 30%–50% nonadherence is likely to be an underestimate. Measurement of all three components of nonadherence and longer follow-up times might demonstrate that nonadherence is more common than adherence in many patient populations.

## 4 Measurement of (non)adherence

There are multiple methods of measurement of nonadherence including self-report, healthcare records analysis, electronic monitoring and biomarker evaluation (discussed elsewhere in this special edition). The consensus remains that there is no universal “gold standard” for medication nonadherence assessment which is universally applicable (Sabaté, 2003) with cross-referencing of multiple methods often identifying more patients who are not adherent. The idea of ‘gold standard’ also varies depending on the concept that is being explored. For implementation, electronic adherence monitors that can capture the time and date of dosing may be the closest to being a ‘gold standard, particularly with some monitors such as digital inhalers that can monitor inhalation (Chan et al., 2013). For medication initiation in an ambulatory setting, pharmacy claims data may be considered the ‘gold standard’ if the patient can only acquire the prescribed medication from a community pharmacy (Rasmussen et al., 2022). However, what may be considered gold standard will depend on the purpose for measuring adherence and there are an ever-increasing range of methods to assess nonadherence, each with their own advantages and disadvantages (Table 1).

**TABLE 1:** Methods to measure nonadherence and their advantages and disadvantages.

	Self-report	Prescribing or dispensing records	Electronic medication monitors
Advantages	• Cheap • Easy to administer • Limited preparation required • Can provide behavioural insights into reasons for nonadherence	• Cheap • Routinely collected • Objective • Can provide information on longitudinal trends and patterns of adherence • Useful for population level analysis • Can be linked easily with other electronic health records	• Granular information on time/date of dosing • Useful for individual level data to tailor adherence strategies to the individual as part of adherence discussions • Can capture diverse range of information as part of predictive analytics • Functions to support adherence • Real-time data can inform early warning alerts
Disadvantages	• Prone to bias • Often only cross-sectional snapshot of adherence	• Requires data cleaning and processing to interpret • Only proxy for medication consumption	• Expensive • Not routinely available • Technical faults possible

### 4.1 Self-report measures of medication nonadherence

Despite the potential for harnessing new technologies to map nonadherence, arguably self-report measures are still the dominant technique used to evaluate the extent to which someone is following the recommendations of their healthcare provider (Kamusheva et al., 2024). Often, they are relatively low cost, can be more feasible to use in routine care, can provide an immediate picture of adherence to facilitate intervention and can give insight into elements of nonadherence that may not be accessible from other measurements.

Common critiques of self-report measurement include that it risks over-estimation due to social desirability bias, is reliant on accurate memory of nonadherence, and may be dependent on patients having an accurate understanding of the recommendations that they have been given about how to take their medication (Stirratt et al., 2015). There is reasonably strong evidence that some patients who self-report good adherence are not accurately reporting their behaviour, for example, a recent US study (Hebel et al., 2020) used urine testing for biomarkers for antiretrovirals for pre-exposure prophylaxis of HIV and found that 12%–15% of patients self-reporting full adherence had nonadherence indicated through urine testing.

The COSMIN checklist (Mokkink et al., 2010), arose from a consensus exercise focusing on how to evaluate patient reported outcome measures such as adherence self-report measures. It highlights internal consistency, content validity, hypothesis testing for construct validity, criterion validity and responsiveness as key dimensions on which to evaluate new measures. These criteria have been increasingly applied to evaluate or develop self-report adherence measures. Kwan et al. (2020) found most self-report measures of (non)adherence had good evidence of construct validity, structural validity and content validity. However, there was weak evidence of the test-retest reliability perhaps unsurprising, given that adherence can be a dynamic behaviour, minimal evidence relating to cross-cultural validity of measures, and poor reporting of how measures how been developed. Tegegn et al. (2022) reviewed self-report measures for medication (non)adherence in cardiovascular disease against the COSMIN criteria; no measure assessed all elements of initiation, implementation, and persistence/discontinuation, with most focused on implementation and none on initiation. Few (non)adherence self-report measures have been validated across all target conditions or groups, or in a wide range of languages/cultures, with implications for relevance. For example, Vianna et al. (2021), reviewed the use of self-report measures to assess adherence to warfarin therapy and highlighted that generic measures had been used but that these did not capture adherence to some of the medication-taking recommendations (e.g., changing dose if experiencing bleeding) which patients taking warfarin are asked to follow.

Overall medication nonadherence self-report measures are increasingly robustly validated. For example, Chan et al. (2020), reported on the development of a five-item self-report scale, the Medication Adherence Report Scale (MARS-5) which included items probing intentional and unintentional nonadherence and considered properties including internal reliability, construct validity and hypothesis testing. The Morisky Medication Adherence Scale (MMAS-8) is another commonly used eight-item structured, self-report measure that assesses medication adherence (Morisky et al., 2008). There are also disease specific questionnaires. Wilson et al. (2016) developed a three-item measure for use in patients with HIV based on reported doses taken/missed over the previous 30 days and validated against objective measures. Dima et al. (2017) validated a measure of nonadherence in asthma against dispensing records and probed overuse of treatment as well as underuse.

With the rise of ‘open science’ and increasing concern for access to scientific tools and outputs, there has been an increasing focus on legal and cost implications of self-report adherence measure use Tesfaye and Peterson (2022) highlight that many measures may be infeasible for clinicians and researchers in low resource settings to use due to legal and cost restrictions. In clinical practice there may be additional barriers of time, and uncertainty regarding whether the measure is validated for use that fits with clinical practice (e.g., verbal delivery). Garfield et al. (2011) highlighted that there is limited available data on key factors relevant for clinicians assessing adherence such as how long a measure takes to complete, and suitability for carer completion.

Selection of a self-report adherence measure therefore needs consideration of the psychometric properties of the measure, the aspects of adherence that need to be assessed, consideration of use restrictions and cost, and the available data on relevance to the particular research/clinical context, patient group and medication. The use of validated self-report measures can therefore be used to provide key insights into the behavioural drivers of medication nonadherence.

### 4.2 Electronic monitoring and technologies to measure nonadherence

As our understanding of nonadherence as a behaviour has advanced, so too have technologies to assess nonadherence. These have been developed to better capture the complexity of nonadherence and medication-related behaviours, whilst at the same time providing opportunities for nonadherence promoting interventions. Electronic medication monitors (EMM) have existed since the 1990s in the form of smart inhalers (Julius et al., 2002), electronic dispensing ‘smart’ pill boxes/bottles and smart pills. These devices in its simplest form record the number of doses taken over time, though current available devices now routinely capture date/time stamps of each medication dose. A recent meta-analysis showed that individuals receiving EMM has significantly reductions in nonadherence with a large magnitude of effect though this did not always translate to clinical benefit in studies which reported both outcomes (Chan et al., 2022). Whilst EMM capture one aspect of medication taking – which is opening of the medication container or inhaler actuation, EMMs still cannot confirm actual medication consumption, which may explain why EMM studies of adherence do not always correlate with clinical outcomes (Chan et al., 2013). How nonadherence relates to clinical outcomes is a question that requires further exploration outside this review but is worth acknowledging that nonadherence alone is only an intermediate outcome and that changes in clinical outcomes are possible even without associated increases in nonadherence.

More sophisticated EMM can also capture location of dosing, allowing linkage with GPS-related data such as environmental factors, and linkage with other datasets. One example is the Propeller Health adherence monitoring inhaler device which can record the location of reliever inhaler use (Merchant et al., 2016). The ability to track and map the location of medication use has provided insights into where ‘hot spots’ of asthma attacks are occurring and allowed further investigation into linkage with environmental triggers such as weather and pollen. This is likely to have important benefits as the effects of climate change increase in years to come, with geographic mapping of medication nonadherence offering new insights to inform resource planning, medication access policies and population health management. EMM can link with wearables, health provider portals, patient apps and be used with AI in predictive analytics to see how changes in patterns of medication use can predict outcomes. For example, changes in reliever medication use alone without input from any other predictors has been shown to predict the onset of an asthma exacerbation 5 days before the attack occurs (Lugogo et al., 2022). The availability of real-time medication use data can thus be used to inform early-warning systems and alerts for patients and providers of negative health outcomes.

### 4.3 Prescription refill database and “big data” analysis of care records

Another method of nonadherence assessment that has exploded in the last 20 years is evaluation of prescription and pharmacy databases to establish patterns of prescription redemption as a proxy for medication-taking. A range of indicators can be calculated. These include whether a prescription is redeemed, indicating primary nonadherence or non-initiation (Cheen et al., 2019). Medication possession ratio whereby the number of doses of redeemed treatment are evaluated against the number of doses prescribed can also be calculated to indicate potential implementation nonadherence in terms of missed doses (Vink et al., 2009). Finally, the date of last prescription refill can indicate discontinuation or persistence with treatment (Gershon et al., 2021).

Prescription refill data has been validated against nonadherence biomarkers and self-report [e.g., (Osula et al., 2022)], with studies showing moderate correlations between medication possession ratio and other outcomes that would be associated with nonadherence (Hood et al., 2018). Unlike self-report data it is ‘objective’ and less likely to be influenced by social desirability bias. But, prescription refill rates are known to be affected by factors such as oversupply, and prescribing duration patterns (Galozy et al., 2020). In addition, prescription refill data can only indicate whether a medication is dispensed but not whether or how it is taken such as timing, storage or use (e.g., inhaler technique), so may not correlate with some nonadherence outcomes (Pattock et al., 2024).

Another potentially useful tool to provide system- or population-level analysis of nonadherence is the utilization of patient records. For example, nonadherence discussions and support provided by healthcare providers and recorded in electronic patient notes may provide insight into patterns of nonadherence (Insani et al., 2023). Healthcare records systems may have specific codes or processes for healthcare professional logging of nonadherence, but this data is yet to be widely used in research. Healthcare records are being linked to pharmacy and other data to gain additional insights (Xu et al., 2023). The growth of large language models and artificial intelligence offer potential for data mining of electronic healthcare and pharmacy records to gain insights into nonadherence (Turchin et al., 2024). Offering nonadherence support automatically to certain patients based on healthcare records has been piloted (Bosl et al., 2013) but is under-explored. As dispensing data are often routinely collected, and, depending on access rights and availability, accessible to healthcare professionals they may be a useful cue for provision of nonadherence support within daily practice. Another use of prescription or dispensing records is to track longitudinal medication use and examine how trajectories of treatment initiation and discontinuation relate to outcomes (Hommel et al., 2017).

## 5 Causes of nonadherence

Nonadherence is widely recognised as a complex behaviour with multifactorial causes (Foley et al., 2021). Kardas et al. (2013) conducted a review of systematic reviews of determinants of nonadherence, highlighting 771 factors that had been linked to nonadherence. This complexity means that for any patient, there are likely to be multiple facilitators and barriers to nonadherence, and that no single intervention is likely to be effective in ensuring nonadherence across all patients, all of the time (Nieuwlaat et al., 2014). Of note, despite the large range of factors identified in the review of reviews, there remained a great deal of unexplained variance in nonadherence behaviour, suggesting that most studies simply cannot test all of the large number of relevant factors that contribute to nonadherence or that untested factors or interactions between factors may drive nonadherence.

To simplify this complexity, there have been classifications of nonadherence determinants. Sabate (2003) stated factors could be patient-, condition-, healthcare system-, therapy-related or socioeconomic, emphasising that causes of nonadherence go beyond individual patients. Several approaches to understanding causes of nonadherence have highlighted factors external to the patient. The Perceptions and Practicalities Approach (Horne et al., 2018), emphasises patients can be nonadherent due to both practical factors, e.g., cost, medicines access, largely leading to unintentional nonadherence and perceptual factors, e.g., beliefs, emotional responses largely leading to intentional nonadherence. Likewise, the COM-B model applied to nonadherence, states patients will not adhere without the physical and psychological Capability (e.g., swallowing capacity, memory), social and physical Opportunity (e.g., support from family, housing) and the reflective and automatic Motivation (e.g., impulses, beliefs) to adhere (Jackson et al., 2014).

Comparatively less focus has been placed on understanding healthcare system or healthcare professional factors that contribute to nonadherence, although the COM-B model could be applied to behaviours of anybody involved in adherence processes including carers and healthcare professionals. The Medication Adherence Contexts and Outcomes Framework (Bartlett Ellis et al., 2023), depicts medication adherence as a series of processes involving different individuals, locations and outcome behaviours. For example, a patient and healthcare provider may interact at a clinic leading to treatment prescription process and the outcome of treatment initiation. Mapping what is known about causes of nonadherence to different processes and individuals involved may enable the development of timely interventions strategies.

All three stages of adherence appear to be strongly influenced by patients’ evaluation of the benefits and harms of medication (Pound et al., 2005). At the initiation stage, the decision to start medication is conditioned by memories of past experiences, environmental influences and preconceived ideas possibly to a greater extent than other stages of adherence (Gil-Girbau et al., 2020; Chapman et al., 2024). At treatment initiation, patients’ emotional reaction to diagnosis and treatment recommendations is key and the health provider-patient relationship appears central to the patients’ experience and decision to initiate the medication (Chapman et al., 2024).

Less is known about factors that affect patients’ decisions to discontinue treatment with most of the published work in this area focused on mental health or cardiac conditions (Keogh et al., 2022). Available studies show that the decision to discontinue medication is often a carefully considered one by the patient, rather than an impulsive action, and is influenced by social, environmental and personal factors (Keogh et al., 2022). Experiences of adverse effects and a desire to regain agency and control have been reported to influence discontinuation (Gershon et al., 2021; Keogh et al., 2022; Gameiro et al., 2012).

## 6 Future directions for research and practice

The WHO states that “adherence is the single most important modifiable factor that compromises treatment outcome” (Sabaté, 2003). With the millions of dollars that are invested yearly into new pharmaceuticals, there is an urgent need to refocus the priorities of clinicians, researchers, funders, and policymakers on addressing nonadherence. Without adherence, there can be no gains made from new healthcare innovations. Yet despite over 50 years of research into nonadherence, the gains that have been made in practice have been minimal. With the new opportunities offered by big data, electronic healthcare databases, digital technologies and AI, the ability to deliver personalised care tailored to the individual’s treatment beliefs, illness perceptions and practical barriers should be a part of routine care. The ability to measure an individual’s beliefs and perceptions via validated questionnaires was one of the major breakthroughs in the last 30 years, allowing quantification of patient experiences without needing to rely on qualitative data (Weinman et al., 1996; Horne et al., 1999). This enables practitioners and researchers to rapidly and accurately assess patients’ beliefs, which should allow the delivery of tailored interventions. Combined with AI that could be used to ‘learn’ from the patients’ responses to questionnaires about adherence and factors driving adherence and the resulting adherence behaviour, there is potential to detect, measure, intervene and potentially predict future nonadherence within the same intervention.

Because nonadherence can change within and between individuals and over time, the continued focus on reporting nonadherence as a static average percentage is likely a further barrier to advancements in adherence research. Early work suggested that for antihypertensive medication a threshold of 80% of medication taken was sufficient to lower blood pressure (Haynes et al., 1980). However, a recent systematic review of (non)adherence thresholds in relation to clinical outcomes found that reported thresholds used to classify nonadherence status range from 46% to 92% (Baumgartner et al., 2018) meaning the validity of the historical 80% threshold could not be confirmed. With the uncertainty in the 80% threshold and the wide variability across patients and conditions associated with this cut-off, it would be prudent for future studies to move away from a static, binary classification system of seeing adherence as a “yes/no” outcome or a simple percentage adherence. Nonadherence needs to be conceptualised as a complex behaviour that requires sophisticated measurement and reporting.

This call for a more personalised, patient-centric approach is captured by Reach in a recent review of nonadherence where he proposes nonadherence should be viewed as a “syndrome” (Reach, 2023). A new model of nonadherence informed by the humanities, philosophy, and behavioural economics is described. The model emphasises the role of character traits, habit-formation, and trust. How this model can be operationalised in practice is yet to be seen. This novel exploration of the intersection between behavioural science and epidemiological methods could hold the key to future solutions for nonadherence.

## 7 Summary and conclusion

In summary, the complexity of adherence is increasingly recognised, leading to the development of new frameworks and approaches to define, measure and understand the causes of nonadherence. Rather than a binary concept, adherence is increasingly being seen as a process that happens over time and involves a wide range of individuals. With this change in conceptualisation, a wider range of measurements and causes are being understood. Rates of nonadherence seem likely to exceed the oft-quoted 30%–50% figure once a full range of aspects are considered. New technologies offer the potential to of a more granular understanding of nonadherence and more effective support for patients taking medicines. There is a need for a paradigm shift for all researchers, clinicians and policymakers to use these technologies to their full potential and to see nonadherence as a health behaviour that shifts and changes with time rather than a static characteristic. Only then can we truly address nonadherence in a personalised, equitable and timely manner.
